# Needs assessment and evaluation of a short course to improve faculties teaching skills at a former World Health Organization regional teacher training center

**Published:** 2015-01

**Authors:** JAVAD KOJURI, MITRA AMINI, ZAHRA KARIMIAN, MOHAMMAD REZA DEHGHANI, MAHBOOBEH SABER, LEILA BAZRAFCAN, SEDIGHEH EBRAHIMI, RITA REZAEE

**Affiliations:** 1 Quality improvement in clinical teaching Research Center, Shiraz Education Center, Faculty of Medical Education, Shiraz University of Medical Sciences, Shiraz, Iran;; 2 Ethics Faculty, Shiraz University of Medical Sciences, Shiraz, Iran

**Keywords:** Faculty, Teaching, Effectiveness, Assessment, Needs assessment

## Abstract

**Introduction:** In the design of educational programs, much attention has been paid to teaching methods, needs assessment, an important part of the development of educational programs, generally is neglected. Another important aspect in educational program design is assessing effectiveness. The aims of this study were to design a formal needs assessment program to define the core contents of a faculty development program, and to determine whether participation in the faculty development program reinforced new teaching skills.

**Methods:** A teacher-training program was designed at Shiraz University of Medical Sciences to help medical instructors boost their teaching skills. Needs assessment was done with nominal group technique followed by a 5-point Likert scale questionnaire. The program, imparted in workshop format, covered effective teaching methods, feedback, assessing knowledge and time management. Instruction was in the form of lectures, group discussions, case simulations, video presentations and role-plays. The program was evaluated in several phases using data triangulation and multi-item assessments of overall program quality in three major dimensions: Kirkpatrick program evaluation model, evaluation of the educational environment and qualitative analysis with open-ended questions. All participants in the study belonged to the academic staff of Shiraz University of Medical Sciences (n=396).

**Results:** Seven main categories were derived from nominal group techniques and questionnaires. After the program, participants rated the quality of the program highly. They felt that the educational intervention was appropriate and had a positive impact on their knowledge of effective teaching methods, feedback, knowledge assessment and time management. Assessment of the effectiveness of the program showed that participants reported significant improvements in their teaching abilities.

**Conclusions:** Our faculty development program  have a significant positive effect on medical university teaching staff members’ competencies. Further research is needed to investigate whether the faculty development program actually results in improved teaching performance.

## Introduction

Faculty members of medical schools play a strategic role in the training and instruction of medical practitioners. Teachers are expected to address a wide variety of educational goals as they work with medical school students. Due to the complexity of effective teaching, medical teachers need to be able to deploy many teaching skills. Planned activities to enhance faculty members’ teaching, administrative and education research skills are known collectively as faculty development (1).

In response to the need for better trained faculty members and with the aim of improving their teaching skills in Iran and the Eastern Mediterranean area, the World Health Organization (WHO) designed an international teacher training program in 1969. Eight centers were selected to train teachers in their own setting. In 1972, the Teacher Training Center at Shiraz University was established and selected as a regional teacher training center (2). This center subsequently designed a model for teacher training at universities in Iran and the Eastern Mediterranean area. In 1996, the center was renamed the Education Development Center and charged with the major responsibility of making academic staff aware of educational processes and teaching skills. The faculty development programs became increasingly popular; however, a systematic approach was needed for needs assessment, planning and evaluation to show whether the programs had any impact on the participants’ practices (2).

In designing educational programs, much attention has been paid to teaching methods such as small group teaching, workshops, e-learning and simulation. A neglected area is needs assessment (3), an important part of educational program development. Previous studies showed that especially in the continuous professional development field, learning will lead to changes in practice when needs assessment has been conducted (4).

Another important aspect of educational program design is assessing program effectiveness. There is little published research that demonstrates the effectiveness of educational interventions. Most studies have relied on indirect measures such as learner satisfaction surveys or self-assessment by participants (5-8).

Accordingly, the Education Development Center of Shiraz University of Medical Sciences (SUMS) developed a needs assessment procedure for use in designing a faculty development module. The faculty development program, which has been running since 2008, comprises a variety of educational methods. The present study aimed to define the process of needs assessment, implementation and evaluation of the effectiveness of the program with a triangulation design. 

## Methods

***Needs Assessment***

This study was prepared in three main stages during 2008: needs assessment, implementation and analysis. The first stage was begun by forming a panel of 20 experts in medical education, and using Nominal Group Technique (9) to determine important topics for inclusion in the faculty development program. The group leader determined the importance of different tasks and objectives. Experts were asked to identify important topics for the faculty development program, and each topic proposed by each expert was recorded. In the second step each topic was discussed in detail to determine its strengths and weaknesses. A list of 7 major important topics and 27 subtopics were developed with this method. This list was used to prepare a questionnaire which was sent to 200 faculty members of Shiraz University of Medical Sciences. For each item, faculty members were asked to indicate their opinion on a five-point Likert scale where:

1) definitely should not be included in the program;

2) probably should not be included in the program;

3)****uncertain as to whether it should be included in the program;

4) important and probably should be included in the program; 

5) very important and definitely should be included in the program.

All topics that obtained a mean score higher than 4 were selected for inclusion in the faculty development program.

***Implementation***

In the second stage, announcements about the program were sent to different departments as posters, banners and information on the university website. Faculty members were also sent emails and sms about the program. To ensure quality, a well-equipped hall with audiovisual support was made available. A budget was considered for the program to cover printing costs, soft and hard copies of medical education journals**,** awards for presenters, accommodations for invited lecturers and refreshments.

 A workshop format was used for most of the program. The flexibility provided by workshops can encourage skill acquisition in faculty development programs and prepare faculty members for new curricular contents (e.g., problem-based learning), and can to help participants familiarize themselves with a new teaching environments (e.g., ambulatory setting teaching).

The Education Development Center of SUMS developed the workshops for academic staff as 1-month medical education programs, called the Medical Education Fellowship. The workshops were held four times per academic year. A core group of faculty provided half-day sessions on topics such as effective teaching skills, curriculum planning, evidence-based medicine, computer and statistical skills, leadership, faculty responsibilities, communication skills, professionalism, interprofessional education, evaluation and feedback, scientific writing, problem-based education, and educational scholarship. The methods of instruction used in the program consisted of lectures which were learner-centered and experiential, with small-group discussion and a problem-based approach. Group discussions were a part of almost every session. Furthermore, video presentations and role-play were used to help participants apply new contents. We tried to provide a friendly environment by creating opportunities for participants to enjoy refreshments and socialize. We offered one credit valid toward academic promotion opportunities for assistant and associate professors, and the program was free of charge. 

A total of 396 faculty member of SUMS from 2008 to 2012 took part in the workshops, of whom 221 (56%) were male and 175 (44%) were female. %52 of the participants were from clinical science faculties and 42% were from basic science faculties. Mean age of the participants was 39 years (range 29-62 years). 

**Program Evaluation**

The program was evaluated in several phases using data triangulation and multi-item assessments of overall program quality in three major dimensions as described below. 

**1. First Dimension (Kirkpatrick Program Evaluation Model)**

The impact of participation on teaching and professional abilities was assessed in three of the four outcome levels (reaction, learning, behaviors, and results) defined by Kirkpatrick (10). To evaluate the first level (K1), participants’ attitudes regarding the effect of training on their educational ability were evaluated with a researcher-administered questionnaire*.* The questionnaire had 10 items that were scored on a 5-point Likert scale (5 = excellent to 1 = very weak) that evaluated the quality of course organization, training materials, program and time management, attainment of course objectives, group interaction, quality of instructors’ presentation style, scientific content, communication with learners and use of educational technology. The questionnaires were distributed to participants at the end of each week.

 In the second level (K2), the effect of the program on participants’ learning was evaluated. The changes in participants’ knowledge were studied with 20-item instructor-administered tests in which each correct response was scored as 1. The tests were given before the training program began. The test instrument covered essential medical education topics taught during the program. The results of this evaluation were compared to those in a control group of faculty members who did not participate in the fellowship program.

In the third level (K3), the participants’ behavioral changes were measured. For this purpose, participants’ teaching style as evaluated by their under- and postgraduate trainees (students, residents) were compared before and after they attended the program with evaluation forms that were designed by the faculty evaluation unit. This evaluation is part of our medical school’s routine quality assurance procedures. Mean scores on this evaluation instrument were compared between participants and a control group.

The fourth level (K4) involved evaluation of the program’s long-term impact on the learners’ career. This evaluation was not done because data collection proved to be complex, time-consuming and costly.

**2. Second Dimension (Evaluation of Educational Environment)**

The educational environment was investigated with a Dundee Ready Education Environment Measure (DREEM) questionnaire in which the items were tailored to the workshop presentations in Persian. The validity and reliability of this 43-item questionnaire were verified previously, and all items were scored on a scale from 0 (strongly disagree) to 4 (strongly agree) (11). The maximum score of 172 in this modified questionnaire indicates an ideal educational environment as assessed by participants. The modified questionnaire consisted of 5 categories: registrars’ perception of learning, perception of course organizers, academic self-perception, perceptions of atmosphere, and social self-perceptions (11).

**3. Third Dimension (Qualitative Analysis with Open-Ended Questions)**

The effect of the program on educational capacities was assessed from participants’ answers to 3 open-ended questions on their general views and feelings about the program, the educational environment, the teachers, and participants’ suggestions for the future. The evaluation methods and tools used are summarized in Table 1.

**Table 1:** Articulation of program evaluation methods across dimensions in a 1-month medical education fellowship at Shiraz University of Medical Sciences

Program evaluation methods and tools Dimensions	Kirkpatrick model			DREEM questionnaire	Open-ended questions
	K1	K2	K3		
	10-item questionnaire	20-item test	Evaluation form		
Impact of course on satisfaction	*	-	-	*	*
Impact of course on learning	-	*	-	*	*
Comparison of learning objectives to learning outcomes	-	-	*	*	*
Educational environment	-	-	-	*	*
General views and feelings	*	-	*	*	*

***Analysis***

To analyze the K1 dimension, we used descriptive statistics (mean and standard deviation). If the mean of any item was more than 60% of the maximum score (i.e., a score of 3), this was considered to indicate an acceptable level of satisfaction. 

For the second and third dimensions (K2 and K3), SPSS 14 software was used for the statistical analysis. The Kolmogorov-Smirnov test was used to verify normality of the data, and if the resulting p value was >0.05, we used parametric statistics. For K2, *t *-tests for paired samples were used to compare pretest and posttest results, and *t *-tests for independent samples were used to compare the results between participants and a control group. For K3, *t *-tests for paired samples were used to compare the mean scores from the evaluation forms before and after the program, and *t *-tests for independent samples were used to compare the results between participants and a control group. Analysis of the modified DREEM questionnaire was based on the mean score calculated for each of the five categories. The participants’ answers to open-ended questions were categorized by grouping similar comments for each question.

The study proposal was approved by the ethics committee of the Shiraz Education Development Center, and all participants completed an informed consent form. The names of the participants were masked throughout the study. 

## Results

The results of the needs assessment exercise are summarized in Table 2. The seven categories that obtained a mean score of 4 or higher were considered the main core curriculum for this 1-month fellowship program.

**Table 2:** Content of a 1-month medical education program in the education fellowship for faculty members of Shiraz University of Medical Sciences

**Subject**	**Title**	**Teaching strategy**
Curriculum planning	How to design a course plan	Interactive lecturing, small group, problem-based learning, homework for writing a course plan and lesson plan, team work
	How to design a lessen plan	
	Spiral curriculum	
	Integration strategy	
	Curriculum revision	
Teaching methods	Large group lecturing	Interactive lecturing, small group, problem-based learning, general lecture, role modeling
	Interactive lecturing	
	Small group teaching	
	Problem based learning	
	Team based learning	
	Bedside teaching	
	Ambulatory teaching	
Assessment and evaluation	Students’ evaluation of teaching	Interactive lecturing, small group, problem-based learning, team work, home work
	Program evaluation	
	Performance assessment	
Research and research in education	The need for research in health care	Small group, problem-based learning, homework with SPSS, approving a proposal for research, active sharing of knowledge in designing an educational research project
	Medical education research	
	How to use SPSS software	
	Scholarship in education	
Communicate, ethics and professional	Communication skills	Interactive lecturing, small group, role modeling, critical incident education, clinical ethical conferences
	Professionalism	
Educational management leadership	How to be a perfect educational leader	Interactive lecturing, large group education, problem-based learning, team-based learning
	Conflict management	
	Strategic management	
Education knowledge and higher thinking skills	Critical thinking	Interactive lecturing, small group, problem-based learning, case presentation, educational film review, critical incident teaching
	Clinical reasoning	
	Evidence based medicine	

The examples of the some of the important sessions highlighting the key messages are the following:

*Bedside and ambulatory teaching:*

 Subjects discussed in these sessions are: optimizing teaching at the bedside and in the ambulatory setting; teaching a multi-level group, e.g. students, interns, resident and fellows; We made video recordings of examples of good and immoral bedside and ambulatory teaching and asked faculties to role play good and bad bedside and ambulatory teaching in role play sessions.

*Presentation abilities and giving good lectures*:

 The skills required for teaching in large groups and interactive lectures are covered in this session. A video sample of an effective lecturer is displayed and after that participants were asked to give a 10 min lecture on topic of their choice. Conversation on the presentations and feedback was done by tutors and participants.

*Evidence based medicine:*

In this module the importance of evidence in making decision, the format of a question, searching strategies, critical appraisal of different articles and decision making was discussed by tutors and was done practically by participants in small group session.

*Clinical reasoning skills*:

At the start of this session, a medical case with goat disease is used to demonstrate the hypothetico-deductive reasoning to reach a diagnosis. Emphasis is on how to involve learners in the analysis of clinical data rather than just recall. After that each of clinical reasoning tests was explained by tutors and a sample of a good clinical reasoning test was shown to participants.

After needs assessment and implementation, fourteen groups of participants took part in the eight, 1-month programs at our center from 2008 to 2012. A total of 396 persons (55% of all faculty members of SUMS) attended the program. 

The results of the K1 analysis showed participants’ overall' satisfaction with the workshops was high (Table 3).

**Table 3:** Participants’ views on the impact of the 1-month medical education fellowship on their educational abilities at Shiraz University of Medical Sciences (Mean scores ranged from 1 to 5.)

Impacts of the program		N	Mean±SD
1	The program consisted of new concepts that can improve my teaching.	391	4.44±0.83
2	The course plan was good.	392	4.25±0.92
3	The program was a good learning experience.	389	4.04±0.89
4	The instruction provided was relevant to my practical responsibilities.	392	4.43±0.65
5	The program scheduling was appropriate.	392	3.36±1.90
6	The program was learner-oriented.	396	4.04±1.13
7	The program reinforced my communication skills.	395	3.98±0.99
8	I feel I have become better prepared in my role as a medical teacher.	389	3.88±0.88
9	The teaching helped to develop my competencies.	390	4.59±0.68
10	The program strengthened my teaching skills and confidence.	396	3.59±0.86

SD=Standard deviation

When we compared pre- and posttest scores in the intervention group to assess changes in knowledge (K2 analysis), we found a significant increase in participants’ cognitive knowledge (mean pretest score 9.23±3.87 out of a maximum of 20; mean posttest score 15.25±3.64 out of 20). The mean posttest score increased significantly in the intervention group (p<0.001), which was evidence of the positive impact of the intervention. Comparison of the posttest scores in the intervention and control groups showed that the difference between groups was statistically significant (p<0.001). Our comparison of baseline characteristics between participants and nonparticipants detected no significant differences in the pretest scores between the control and intervention groups (p>0.05). 

To assess behavioral changes and the application of learning in the workplace (K3), we compared participants’ and nonparticipants’ ratings by their under- and postgraduate trainees before and after the program. A total of 2500 trainees rated 396 participants in the intervention group and 360 faculty members in the control group. There was no significant difference between the under- and postgraduate trainees’ evaluation of teaching effectiveness between the participant and control groups before the intervention (p=0.78). The mean rating for faculty members who participated in the program (18.76 out of 20) was significantly higher (p<0.001) than their rating before the course (17.68 out of 20). No such improvement was seen in the control group’s rating, indicating the positive impact of the program on teaching effectiveness 

Evaluation of the educational atmosphere with the DREEM questionnaire yielded a mean score of 156.51 (90.9%) out of a possible total of 172 across five educational subscales. This was evidence of the excellent educational climate during the program sessions. 

The mean score on the perception of learning subscale was 46.2±7.55 out of a maximum score of 48 (teaching highly thought of), and mean score on the course organizer subscale was 35.69±7.02 out of a maximum of 40 (model course organizer). For the perception of atmosphere, the mean score was 25.66±5.62 out of 32 (good feeling overall). On the academic self-perception subscale, the mean score was 22.87±5.02 out of 24 (confident), and on the social self-perception subscale, the mean score was 23.23±4.88 out of 28 (very good socially). The mean scores for each subscale are summarized in Table 4.

**Table 4:** Total and subscale scores based on the DREEM questionnaire of participants’ perceptions regarding the educational atmosphere in the 1-month medical education fellowship at Shiraz University of Medical Sciences

Educational subscale		N	Number of items	Max	Mean±SD
1	Perception of learning	390	12	48	46.2±7.55
2	Perception of course organizers	396	10	40	35.69±7.02
3	Perceptions of atmosphere	394	8	32	28.52±5.99
4	Academic self-perception	390	6	24	22.87±5.02
5	Social self-perceptions	391	7	28	23.23±4.88
Total			43	172	156.51±6.22

Our analysis of the participants’ responses to open-ended questions showed that most participants thought the overall course quality was good. According to most participants the program increased their motivation to acquire teaching and communication skills. Some of their free comments on the program’s strengths noted that there was good communication between them and the instructors, the program objectives were clear, and that group work and interactions were satisfactory. Some of them noted that they enjoyed of the program and appreciated the friendly climate. 

## Discussion

Academic vitality is dependent on faculty members’ expertise, and faculty development plays an important role in promoting academic excellence. However, faculty development programs needs to be systematic, and require coordination and planning, implementation and assessment(12, 13). Investments in time and effort to develop teaching skills are necessary to help faculty members succeed in their various roles, to improve program management, and to make better use of time. Instructors need to design practical methods for measuring expected outcomes in order to determine the effectiveness of faculty development programs (14).

Needs assessment is vital before changes are undertaken in teaching content or educational strategies. In descriptions of adult education, Knowles assumed that adult learners desired to sense a need to learn, and identifying one’s personal learning requirements was an important part of self-directed learning (4). The present study reports a good example of needs assessment in designing an education program for faculty development. However, we are aware that one of the limitations of this part of our study is that formal needs assessment can identify only a constricted choice of priorities and may miss other needs.

The primary assumption of faculty development is that it will eventually serve the critical goals of medical education, i.e. improving patient and community care by educating qualified medical practitioners. The outcomes of faculty development are an important issue; despite several decades of reported success with faculty development programs, little has been published on their outcomes (14). The literature commonly reports outcomes as short-term gains in knowledge, changes in attitudes, satisfaction with the program, and self-reports of changes in behavior (15, 16). Many of these published studies, however, lacked a control group—a shortcoming that may call their findings into question (14). Moreover, some previous studies did not make use of mixed modeling (17). Our triangular analysis shows that the program we developed achieved many of its stated educational objectives. The evaluation results were positive and showed statistically significant improvements in participants’ teaching and evaluation skills, whereas no such improvements were seen in nonparticipants, as judged by their under- and postgraduate trainees.

Our assessment of the results for the K1 dimension showed that participants reported high satisfaction with both specific topics and the overall program. The results of a systematic review of 53 faculty development programs found that such programs resulted in high levels of satisfaction and changes in attitudes, knowledge, skills, and teacher behaviors (11).

In the K2 dimension, the pretest and posttest results showed that there were significant increases in participants’ knowledge, and significant differences between the case and control groups. Gains in knowledge and skills were also reported in 53 other faculty development programs (18).

In the K3 dimension, we obtained students’ evaluations of teachers before and after participation in our program, and compared both evaluations to those students provided for nonparticipants. The results were positive and showed statistically significant improvements in participants’ teaching and evaluation skills, but no such improvements in nonparticipants, as judged by under- and postgraduate trainees. This result is evidence of the positive impact of the program on teacher effectiveness. 

The educational environment of our program was rated favorably by participants in a modified DREEM questionnaire. This result suggests that careful preparation of the teaching sessions, appropriate feedback from instructors and the creation of a suitable environment can improve the quality of teacher training programs. The World Federation for Medical Education has indicated that the learning environment is one of the targets for the assessment of educational programs (19). The DREEM questionnaire is a reliable and valid instrument that is not culture-specific, and the positive results of our study strongly support the efficacy of the educational environment created for our educational program (20).

In qualitative, open-ended questions the quality of our program was rated well by participants. A positive attitude, beliefs and motivations were, as noted above, areas of concern for our program managers. According to the education literature, motivation influences the learning process and outcomes (21). Our program may motivate participants, since they reported a need for similar programs in the future.

Because the work schedule of our participants changed during the training period, we faced challenges in ensuring faculty members’ participation, and this led to an element of resistance among some participants—an issue that should be addressed by the program managers. Some participants could not attend certain sections of the program and commented on specific items for inclusion in the program. In these cases we tried to accommodate their requests by organizing additional sections which could be implemented by small-group teaching. However, the most important aspect of managing these instances of minor resistance was our emphasis on motivating participants and using new educational techniques.

According to all dimensions we evaluated, the program had a positive impact on teaching abilities. The results of the present study support the findings of previous studies that examined faculty development programs (2, 11, 19, 22, 23). Although others have previously reported positive outcomes of teacher training intervention programs, our findings are important because we used data triangulation and multi-item assessments for our evaluation exercise, included an age- and sex-matched comparison group, and implemented a multi-item triangular design to increase the validity and reliability of our findings. However, the fourth level of effectiveness (the program’s long-term impact) could not be evaluated because of the prolonged time frame needed to obtain longitudinal data on the influence of our training program on faculty members’ career. The broader effects of our educational intervention on changes in teaching practice should thus be documented in further research.

Taken as a whole, our findings demonstrate that a faculty development program can improve teaching skills. The approach we used was informative, and aimed to determine course strengths and weaknesses as well as to develop practical recommendations for improvement.

**Study limitations**

We were unable to analyze the fourth level of effectiveness due to time and administrative constraints. Participants took part in the fellowship program voluntarily, but their motivation may have been influenced by the potential competing interest of credit gained toward academic promotion. This factor is sometimes considered an element that “obliges” faculty members to participate in workshops, although enrolment in the study was completely voluntary and none of the attendees refused to participate in the present analysis. Longitudinal and cohort studies are recommended to measure to long-term impact of faculty development programs. 

## Conclusion

This study presents evidence of the efficacy and usefulness of a faculty development program designed to increase teachers’ knowledge of the principles and philosophy of education based on a well designed needs assessment process and careful implementation. We show that there was a statistically significant improvement in teaching effectiveness, with a positive, medium-term gain in skills as a result of our faculty development program. Nevertheless, retention of the new teaching skills acquired through the program decreased with time. Further studies are needed to determine whether booster programs might help faculty members maintain appropriate teaching skills in the medium and long term. Clearly, faculty development is an important aspect of medical education. We concur that faculty development will promote the professionalization of teaching and must be an essential aspect of every medical school; therefore it is our duty to design formal approaches to achieve our goals in this area. 
